# Nutritional approach for increasing public health during pandemic of COVID-19: A comprehensive review of antiviral nutrients and nutraceuticals

**DOI:** 10.34172/hpp.2021.17

**Published:** 2021-05-19

**Authors:** Vahideh Ebrahimzadeh-Attari, Ghodratollah Panahi, James R. Hebert, Alireza Ostadrahimi, Maryam Saghafi-Asl, Neda Lotfi-Yaghin, Behzad Baradaran

**Affiliations:** ^1^Department of Nutrition and Food Sciences, Maragheh University of Medical Sciences, Margheh, Iran; ^2^Department of Clinical Biochemistry, Faculty of Medicine, Tehran University of Medical Sciences, Tehran, Iran; ^3^Department of Epidemiology & Biostatistics, Arnold School of Public Health, University of South Carolina, Columbia, South Carolina, USA; ^4^Cancer Prevention and Control Program, Arnold School of Public Health, University of South Carolina, Columbia, South Carolina, USA; ^5^Nutrition Research Center, Department of Clinical Nutrition, Faculty of Nutrition & Food Sciences, Tabriz University of Medical Sciences, Tabriz, Iran; ^6^Student Research Committee, Department of Clinical Nutrition, Faculty of Nutrition & Food Sciences, Tabriz University of Medical Sciences, Tabriz, Iran; ^7^Immunology Research Center, Tabriz University of Medical Sciences, Tabriz, Iran

**Keywords:** COVID-19, Coronavirus, Immunity, Energy intake, Proteins, Fatty acids, Omega-3 Vitamins, Vitamins, Minerals, Electrolytes

## Abstract

**Background:** The novel coronavirus (COVID-19) is considered as the most life-threatening pandemic disease during the last decade. The individual nutritional status, though usually ignored in the management of COVID-19, plays a critical role in the immune function and pathogenesis of infection. Accordingly, the present review article aimed to report the effects of nutrients and nutraceuticals on respiratory viral infections including COVID-19, with a focus on their mechanisms of action.

**Methods:** Studies were identified via systematic searches of the databases including PubMed/ MEDLINE, ScienceDirect, Scopus, and Google Scholar from 2000 until April 2020, using keywords. All relevant clinical and experimental studies published in English were included.

**Results:** Protein-energy malnutrition (PEM) is common in severe respiratory infections and should be considered in the management of COVID-19 patients. On the other hand, obesity can be accompanied by decreasing the host immunity. Therefore, increasing physical activity at home and a slight caloric restriction with adequate intake of micronutrients and nutraceuticals are simple aids to boost host immunity and decrease the clinical manifestations of COVID-19.

**Conclusion:** The most important nutrients which can be considered for COVID-19 management are vitamin D, vitamin C, vitamin A, folate, zinc, and probiotics. Their adequacy should be provided through dietary intake or appropriate supplementation. Moreover, adequate intake of some other dietary agents including vitamin E, magnesium, selenium, alpha linolenic acid and phytochemicals are required to maintain the host immunity.

## Introduction

The global outbreak of coronavirus disease 2019 (COVID-19) has reached pandemic proportions, heightening public health concern around the world. Coronaviruses (CoVs), the single-stranded RNA viruses, have large, positive and enveloped senses that can affect both humans and animals.^1^

COVID-19 is the third serious coronavirus outbreak in the world following severe acute respiratory syndrome coronavirus (SARS-CoV) and Middle East respiratory syndrome coronavirus (MERS-CoV). According to the latest statistics of World Health Organization (WHO), there are more than 99 million confirmed COVID-19 cases and more than 2 000 000 deaths due to COVID-19 around the world.^2^

Clinical manifestations of COVID-19 range from mild symptoms to critical illness and death.^3,4^ Frequently reported symptoms of infected patients include fever (77%–98%), cough (46%–82%), myalgia or fatigue (11%–52%), and difficulty of breath (3%-31%) at the onset of illness, which can progress to severe lung injury and respiratory distress syndrome.^3,5-7^

Several host-related factors can affect the pathogenicity of COVID-19 including age, genetics, immunity, psychosocial status and especially existing comorbidities. However, another important variable is nutritional status, usually ignored in the epidemiological studies.^8^ It was well-acknowledged that malnutrition; e.g., specific dietary deficiencies or severe weight imbalance (including both underweight and overweight/obesity) may affect the consequences of viral infections.^9-16^ Actually, the idea that malnutrition makes a subject prone to viral infection first emerged in the West about 200 years ago.^17,18^ Researchers indicate that host nutritional status not only affects the immune function of the body, but also may have some direct effects on the viral genome and its virulence.^18^ Therefore, assessment of the host nutritional status before and during an infectious disease is of important value that might influence the recovery rate.

Regarding the global pandemic of COVID‐19, preventive policies such as applying public health principles and nutritional support are crucial at this time. Therefore, the present review article aimed to outline nutritional support for the prevention and treatment of COVID-19, focusing on the role of energy, macronutrients, and antiviral micronutrients and nutraceuticals.

## Materials and Methods

Searches were performed in PubMed/MEDLINE, ScienceDirect, Scopus, and Google Scholar databases from 2000 until July 2020 using the following keywords based on MeSH terms. Generally, a topic-centric search was conducted to write each section. All relevant clinical and experimental studies published in English were included. The search terms included “immunity” OR “ viral infection” OR “ COVID-19” AND “nutrition” OR “antioxidant”; “vitamin D”; “vitamin A”; “vitamin C”; “folic acid”; “zinc”; “omega 3”; “protein”; “energy”; “probiotic”; “prebiotic”; “phytochemical”. These search items were used for the relevant sections of the review. Data were extracted from the included manuscripts by one author (MSA). Two other authors checked the accuracy of the data extracted (NL, VEA).

### Energy

Protein-energy malnutrition (PEM), which is associated with low body weight and loss of both lean body mass and adipose tissue is characterized as “the most common cause of immunosuppression worldwide”.^9^ It was reported that if PEM is accompanied by wasting, which is often the case, it can decrease the function of natural killer (NK) cells in both humans and mice^14^ and increase the mortality rate in influenza-infected mice.^19^

Generally, bacterial and viral infections are usually associated with increasing metabolic rate and energy requirements along with anorexia which exacerbates this energy demand.^9,20^ During the COVID-19 pandemic, it has been reported that most of the infection-related mortality occurs in the elderly patients mainly due to malnutrition and other comorbidities.^21^ Although aging is associated with a slight decrease in total lymphocytes number and some changes in T-cell response,^22-24^ this higher mortality rate due to COVID-19 may be justifiable by the presence of PEM.^22^

In this regard, energy requirement of an infected person with COVID-19 increases slightly, like any other viral infection especially during the acute phase of the infection.^9,25-28^ It is ideal to measure the energy expenditure using the doubly labeled water as a gold standard method.^29^ However, this is not practical in all settings and it is generally recommended that energy requirement be calculated carefully and individually, based upon a slight increase during the fever and acute respiratory distress as there is a hyper-metabolic state in both conditions.^27,28^

Current energy recommendations during lower respiratory infections and acute respiratory distress are 30 to 35 kcal/kg ideal body weight (IBW) or resting energy expenditure (REE) ×1.2-1.4 (kcal). Fever also increases REE by approximately13% for each degree above 37°C.^27,28^ It should be mentioned that regular nutritional assessment of hospitalized patients is very important to prevent the consequence of over-feeding (e.g., CO2 overproduction and fatty liver) or under-feeding (e.g., increasing morbidity and mortality rate and pulmonary cachexia).^27,28^

If the measurement of VCO2 is not possible directly, body weight is used for the estimation of energy requirements, as below: 25–30 kcal/kg/d for non-obese and 21 kcal/kg/d for overweight, 11–14 kcal/kg/d for obese critically ill patients.^30^

The adequacy of food intake should be thoroughly assessed in hospitalized patients with COVID-19 pneumonia.^27,28^ Parenteral nutrition (PN) should start if enteral feeding is not possible or adequate. On the other hand, the worldwide outbreak of COVID-19 can be unintentionally accompanied by increasing anxiety, overeating and inactivity in the societies which cause people at risk of obesity. It was reported that obese people are at higher risk of developing either respiratory tract or urinary tract infections, compared to the normal weight people which highlights the important of physical fitness.^15^ Therefore, as a prevention strategy, it is recommended that people avoid the excessive energy intake and ensure sufficient nutrients supply through healthy food preferences. Overall, avoiding under- nutrition or over- nutrition seems to be essential in case of COVID-19 prevention or treatment.

### Macronutrients

It was shown that the amount and kind of macronutrients can change the number and function of immune cells in response to bacterial or viral agents.^31^ The other mechanism for their modulation of immune function may be through the changes in the gut microbiota.^32^ The roles of each macronutrient in immunity are presented in detail, as below:

### Protein

Regarding the hyper-metabolic feature of severe respiratory tract infections like COVID-19, restricting the protein intake can lead to malnutrition which adversely affects lung structure, elasticity, and function.^27,33^ The amount of protein intake can also directly impact the immune system. Based on the results of some experimental studies, feeding a low-protein diet, followed by infection with influenza virus was accompanied by decreased number and function of CD8+ T cells as well as NK cells, higher viral titers, and finally, increased mortality of animals.^34-36^ It should be noticed that older people are at higher risk of protein deficiency^37-41^ which may make them prone to longer hospitalization due to the infection.^41^ Therefore, appropriate protein intake of 1.2 to 1.5 g/kg IBW (15%-20% of calories) with at least 50 percent from high biological value (HBV) proteins is recommended for adults and older people to maintain their respiratory muscle strength and support immune function during infection with COVID-19.^26,27^ However, diet should be individually planned, considering other comorbidities of patient.

According to American Society for Parenteral and Enteral Nutrition (ASPEN), for critically ill patients with COVID-19 who need enteral nutrition (EN), the recommended protein intake is 1.2–2.0 g/kg/d for non-obese patients (based on actual body weight) and 2-2.5 g/kg/d for obese patients (based on IBW).^42^

### Carbohydrates

According to limited animal studies,^32,43^ significant restriction of carbohydrates and sugar intake can exacerbate viral outcomes. This happens because the immune cells like CD4+ and CD8+ T cells, involved in the pathogen clearance, supply most of their energy from glucose and anaerobic glycolysis.^44^ On the other hand, a high-carbohydrate diet increases CO_2_ production and respiratory quotient. Therefore, a balanced ratio of carbohydrates intake (40% to 50% of calories), mostly as the complex carbohydrates along with decreasing simple sugar is crucial in medical nutrition therapy of COVID-19 pneumonia. In addition, adequate intake of functional fibers (prebiotics such as beta-glucan and fructo-oligosaccharides) from dietary sources (e.g., oat, wheat, banana, onions, garlic, and tomato) has additional health benefits on the gut microbiota and immune defense system.^45^

For those with severe COVID-19 symptoms, supportive nutrition may be required. EN is preferred over PN and is usually well-tolerated by the patients. The amount of carbohydrate in enteral feeding formula is dependent upon patient’s state (e.g., glycemic control, ventilator dependency) and usually consists 30%-50% of non-protein calories.^46^ However, glucose should not exceed 5 mg/kg/min.^47^

### Fats

New coronavirus can cause severe lung injury and acute respiratory distress syndrome (ARDS).^48^ During this acute-phase infection, increasing dietary fat intake helps to decrease CO^2^ production as well as ventilator dependency. According to the dietary recommendations for ARDS and pneumonia, an enteral feeding with 30% to 45% of calories from fat maybe helpful.^27,28^ However, the type of fatty acid is very important in the inflammatory and immunomodulatory responses of host to a microbial agent.^49,50^

Adequate intake of omega-3 fatty acids may protect host against viral and bacterial infections. ^51^ However, there are some controversial data about their efficacy in different bacterial and viral infections.^52-55^ A recent review article on the current experimental studies and clinical trials reported that intake of long-chain omega-3 fatty acids as docosahexaenoic acid (DHA) and eicosapentaenoic acid (EPA) was effective in some extracellular infectious agents including Escherichia coli and Streptococcus pneumonia while had adverse effects on the intracellular infectious agents like Mycobacterium tuberculosis, Influenza virus and Herpes simplex virus. It was due to the modulation of immune responses including anti-viral CD8+ T cells and their downstream inflammatory cascades after viral infection.^51,55,56^ Therefore, the efficacy of omega-3 long-chain omega-3 polyunsaturated fatty acids (PUFAs) on defense against viral infections is still unclear and needs further well-designed randomized clinical trials.

The other key element in medical nutritional management of COVID-19 infection with severe lung injury and ARDS symptoms is surfactant replacement therapy.^57,58^ Dipalmitoyl-phosphatidylcholine (DPPC) is the main component of pulmonary surfactants which consists of two palmitic acids, attached to a phosphatidylcholine (lecithin) head-group.^58^ Therefore, in addition to the exogenous surfactant replacement therapy, enteral feeding with dietary sources of lecithin, including egg yolk, meat, milk, and canola oil can be helpful in ARDS.^59^

The amount of fat in EN formula depends on patient’s status (e.g., serum triglyceride, ventilator dependency) and usually comprises 50%-70% of non-protein calories.^47^It was reported that long-term deficiency of essential fatty acids (EFAs) including linoleic acid and ɑ-linolenic acid (ALA) can cause the reduction of DPPC level in lung tissue.^60^ However, based on two recent systematic reviews, there are some controversies toward the benefits of long-chain omega 3 fatty acids supplementation on the length of mechanical ventilation and mortality rate in adult patients with ARDS.^61,62^ Overall, adequate intake of EFAs and appropriate proportion of PUFAs and monounsaturated fatty acids (MUFAs) intake within the range of 30% to 45% of calories from fats can be effective in patients with COVID-19 pneumonia and ARDS symptoms.^63^

### Micronutrients

***Vitamin D***

Vitamin D (VitD), also termed cholecalciferol, has a lot of functions in the body including the immunomodulatory effect.^64^ The deficiency of the vitamin is more prevalent around the world.^65^ Results of some review articles show that low circulating levels of VitD are related to increased risk of respiratory viral infections.^65-67^ However, there are some other heterogeneous findings on the protective effects of VitD supplementation for respiratory tract infections like influenza virus.^65,68,69^

It is established that VitD (cholecalciferol) has an important role in the transcription of different genes through binding to the nuclear VitD receptors (VDRs).^70^ VDRs are expressed by many immune cells, following an immune signaling. Their bindings to VitD result in the modulation of both innate and adaptive immune responses, B-lymphocytes, monocytes, macrophages, dendritic cells (DCs), and T-cells. This immunomodulatory effect on T-cells causes the suppression of the pro-inflammatory Th1 and Th17 cells as well as enhancement of regulatory T cells.

Increased activation, production, and secretion of pro-inflammatory cytokines and chemokines like nuclear factor kappa-light-chain-enhancer of activated B cells (NF-κB) are the main feature of most respiratory viral infections.^71^ Accumulating evidence proposes that a subgroup of patients with severe COVID-19 might have a cytokine storm syndrome.^72^ It was reported that one of the main immunomodulatory effects of VitD against respiratory viruses is increasing the NF-κB inhibitor alpha (IκBα) expression.^73^ Moreover, the active form of VitD (1, 25(OH)2 D3) can decrease the expression of some cytokines including tumor necrosis factor alpha, interleukin 6 (IL-6), and interferon beta (IFN-β) and increase the production of IL-37 and human beta defensin 2 which have key roles in warding off respiratory viruses.^70,74^

VAD is linked to more inflammation and immune activation and low peripheral blood CD4+ T cells.^75^ Low levels of VitD in calves have been correlated with increased bovine coronavirus infection.^76^ Moreover, it was suggested that VAD can increase the host susceptibility to influenza viral infection.^77-79^ It should be noticed that infection with influenza virus, the novel coronavirus, and SARS-CoV have been more abundant in winter, when the circulating level of VitD is the lowest.^77,80,81^

Angiotensin (Ang) II-converting enzyme (ACE2) molecule was reported to be the main host cell receptor of COVID-19 and plays a critical role in the entry of virus into the cell to trigger the final infection.^82,83^ And, it is closely associated with ARDS,^84^ possibly due to an extremely large number of ACE2-expressing cells in the lung, especially in Asian males.^83^ More interestingly, chronic VitD deficiency may induce lung fibrosis through the activation of the renin-angiotensin system.^85^

Therefore, VitD supplementation seems to inhibit ACE2, consequently, helping to attenuate the lung infection. Overall, regarding the limited dietary values, it seems that VitD supplementation, aside from its wide-spread immunological functions, can be considered both as a preventive strategy and an anti-ACE2 therapeutic agent in the management of COVID-19 infection to maintain its serum level at normal range, but avoid the vitamin toxicity.

***Vitamin C***

Vitamin C (VitC), also known as ascorbic acid, is an antioxidant and free radical scavenger and serves as an important cofactor for many enzymatic reactions in the body.^86^ Severe VitC deficiency leads to scurvy disease, marked by weakness of collagen proteins, poor wound healing, impaired immunity, and increased susceptibility to infections.^87^ Studies have shown that VitC can affect the immune system by inhibition of oxidative stress generated by infections.^88,89^ In addition, it has immune-modulating effects including phagocytic function, T-cells transformation and interferon production.^90^ Clinical trials have shown that treatment by VitC reduced duration and severity of common cold episodes.^91,92^ The latest research found that though treatment by VitC in patients with sepsis and ARDS did not improve the organ dysfunction, inflammation and vascular injury. However, mortality rate was significantly decreased.^93^

Leukocytes such as neutrophils and monocytes accumulate VitC and have 50- to 100-fold higher concentrations than plasma, indicating its significant functions in the immunity.^94^ As VitC attenuates both oxidants generation and NFкB activation in dendritic and neutrophils,^95^ the deficiency of the vitamin can result in impaired immunity and higher susceptibility to infections. In turn, infections significantly impact on VitC levels due to enhanced inflammation and metabolic requirements.^96^ Moreover, VitC deficiency may affect the ability of neutrophils to migrate to the sites of infection.^97,98^

Patients with severe respiratory infections have lower plasma VitC concentrations and treatment by VitC restores the plasma VitC levels and ameliorates the severity of the respiratory symptoms.^99^ Additionally, several studies on guinea pigs have demonstrated that supplemental VitC increases serum levels of C1q complement proteins^100-102^ as well as antibodies.^103,104^ However, no beneficial changes in leukocyte function or production have been reported by VitC treatment.^105-107^

While some studies propose VitC as an immune enhancer, human studies published to date are contradictory. According to a Cochrane systematic review, only two of six trials were double-blind, placebo-controlled, and randomized controlled that could be applied to evaluate the effect of VitC supplementation on the prevention or treatment of pneumonia.^108^ Overall, compared to placebo, VitC supplementation had a mild beneficial effect. However, firm conclusions cannot be elicited from the available data due to the highly variable characteristics of the study populations and methodological shortcomings. A phase II clinical trial is underway by Peng et al (https://www.clinicaltrials.gov/) from Wuhan to test the efficacy of VitC infusion for the treatment of severe acute respiratory infection associated with the novel coronavirus. They aimed to administer VitC (24 g/d IV) for 7 days to the afflicted patients.

It seems that VitC can help to avoid and cure respiratory and systemic infections via the enhancement of the immunity. At this time, the potential effect of VitC on severe viral respiratory tract infections especially in case of the recent COVID-19 epidemic would seem to be evaluated by further studies.

***Vitamin A***

Vitamin A (VitA) is one of the fat-soluble retinoids which deficiency is one of the most common nutritional issues in the world.^109^ This vitamin has a broad range of immunological functions. VitA deficiency (VAD) is associated with recurrent infections.^110^ Since VitA helps maintain the mucosal barriers of the innate immune system. Thus, VAD increases susceptibility to some types of infection, such as respiratory and gastrointestinal infections.^111-114^ VAD also results in decreased or altered response of T and B cells to a variety of pathogens; dysregulated development of type IFN; and changes in the number and function of innate immune population, including monocytes, NK cells and DC subsets.^115,116^ Serum retinol is inversely associated with serum concentration of interleukin-6, the main regulator in the induction of the acute-phase response.^117^Infections can, in turn, lead to VAD by reducing food intake, impairing vitamin absorption, increasing vitamin excretion, interfering with vitamin utilization, or increasing metabolic requirements of VitA.^111^

Genetic studies indicated significantly impaired inflammatory milieu in the infected lungs of VAD calves, with alterations in Th1 and Th17 immune responses, and abnormal mucin production. In chickens fed a diet slightly deficient in VitA, the risk of infection with the infectious bronchitis virus, a kind of coronavirus, was more noticeable than in those fed with a VitA-adequate diet.^118^ VitA has been shown to be effective in measles-associated pneumonia in children.^119^ However, high-dose vitamin A supplements caused modest adverse effects in children recovering from pneumonia.^120^ Therefore, they should not be therapeutically used in such patients unless there is a clinical evidence of VAD.

VitA supplementation was studied as a potential intervention to accelerate recovery, minimize severity, and avoid recurrent episodes of acute infections of the lower respiratory tract.^121,122^However, it is not beneficial in those with lower respiratory infections, such as pneumonia ^123^ and supplementation may actually aggravate the condition.^120,124,125^ As VitA can inhibit viral replication through the up-regulation of the innate immunity,^126^ it seems that VitA might be promising in the prevention and treatment of lung infections stemmed from COVID-19. However, due to potential adverse effects, VitA supplements should be administered for those with evidence of VAD.^127^

***Vitamin E***

Vitamin E (VitE), as an antioxidant, protects membranes from oxidative damage by incorporating into cell membranes.^128^ The α-tocopherol form of VitE protects against peroxidation of PUFAs which can potentially bring about abnormal immune responses.^129^ Therefore, VitE is one of the most effective nutrients known to modulate immune function and reduce the risk of respiratory infections and asthma. Owing to its ability to bind free radicals, VitE plays an important function as an antioxidant.^130,131^Immune cells are enriched in VitE, likely to protect membranes against oxidative damage produced as a result of their high metabolic activity and defensive function.^132^

Animal and human studies have demonstrated that VitE deficiency impairs both innate and adaptive immunity.^133^ It leads to immune system dysfunction and there is evidence that VitE supplementation, beyond current dietary guidelines, enhances innate immune functions including NK cell activity and macrophage phagocytic capacity.^134^ VitE may also exert its influence by modulation of inflammatory mediators like prostaglandin E2 and cytokines.^132^

In a trial, daily supplementation of 200 mg of α-tocopherol for 235 days to healthy older adults increased the production of antibodies in response to tetanus and hepatitis B vaccines and improved T lymphocyte-mediated immunity.^135^ In another interventional study, supplementation with 200 mg/d of α-tocopherol for three months to older adults significantly enhanced mitogen-induced lymphocyte proliferation and interleukin-2 (IL-2) production and improved NK cytotoxic activity, neutrophil chemotaxis, and phagocytic response, compared to baseline.^136^ Daily supplementation of 617 nursing-home residents (≥65 years of age) with 200 IU of synthetic α-tocopherol for one year significantly lowered the risk of affliction with upper respiratory tract infections, particularly the common cold, but had no effect on lower respiratory tract (lung) infections.^135,137-140^ Though studies have reported immune effects at dosage of 200–800 mg/d,^141^ the optimum intake of VitE required to enhance immune system has not been demonstrated, likely due to prior VitE status and the presence or absence of other conditions.^142^

***Vitamin B12***

Vitamin B12 (VitB12 or cobalamin) acts as a human immunity modulator; it stimulates the T-lymphocytes production involved in cellular immunity, restores the atypically increased ratio of CD4/CD8 and retains the lymphocyte subgroups count in the adequate range.^141,143^ It supports both antibody-mediated and cellular immunity.^144^ VitB12 deficiency reduces the lymphocytes and CD8+ cells number and the CD4 cells proportion, leading to an unusual high ratio of CD4+/CD8+, immune defense depression against bacteria and viruses and repressed NK cells.^143^

Adequate vitamin dosage in deficient individuals leads to increased percent of CD3 and CD7 cells and their absolute number. Additionally, the function of lymphocytes and NK cells are restored, and IgA, IgG, and IgM concentrations are improved after treatment^144^ Adults (aged >65 years) with normal immunity but with low serum VitB12 had an abnormal antibody response to pneumococcal polysaccharide vaccine.^145^ Supplementation with VitB12 along with folate and VitE in the elderly people increased NK cell cytotoxic activity.^146^ These few studies show the importance of a sufficient VitB12 status to maintain an adequate immune response, especially in the elderly who have a high percentage of deficient serum VitB12 level.^147^

***Vitamin B9 (folate)***

Folate (VitB9 or folacin) along with vitamins B6 and B12 plays a vital role in the synthesis of nucleic acid and protein. Insufficient amount of folate leads to considerable alteration in the immune response.^144,148^ In folate deficiency, the response of antibody to different antigens is reduced, since it results in lower proteins level involved in immune function activation and regulation, whereas a sufficient proportion with adequate pro-inflammatory cytokines supports an efficacious immune response.^149,150^ It was demonstrated that the administration of folate supplements to the elderly improves overall immune function by alteration of the age-associated decrease in NK cell activity supporting a Th1 response; thus, providing protection against infections.^151^Interestingly, the results of a most recent study have proposed folate as a potential compound in the prevention or control of COVID-19 especially in the early stages of disease. In fact, this is the first time that has introduced folate as an effective inhibitor of furin enzyme activity.^152^ The furin protein is associated with increasing the pathogenesis of most of bacterial and viral infections. It was proposed that the entrance of coronaviruses into cells is mediated through the spike proteins (S proteins) on their surface after cleavage into the S1 and S2 domains by furin enzyme. It should be mentioned that after this cleavage, the S1 subunit binds to the ACE2 receptor, the main host cell receptor of COVID-19, and then enters the lung cells. Therefore, supplementation with folate can be considered as a safe and promising treatment for patients with COVID-19.^152^

***Vitamin B6***

Vitamin B6 (VitB6) or pyridoxine has a very important role in general cellular metabolism.^153,154^ Cytokines and antibodies are made up of amino acids and need vitamin B6 as a coenzyme in their metabolism; therefore, the effects of VitB6 on immune function cannot be overlooked.^144,155^ In previous studies, it was revealed that VitB6 deficiency impairs the maturation and growth of lymphocytes, and the production of antibodies and T-cells activity. The mitogenic response of lymphocytes is weakened by depletion of dietary VitB6 in elderly subjects and restored by VitB6 administration. Decreased antibody delayed-type hypersensitivity (DHT) response, NK cell activity, IL-1β, IL-2, IL-2 receptor and lymphocytes proliferation were observed due to deficiency in VitB6 levels.^156-158^

Overall, further studies are warranted to evaluate the efficacy of VitB complex intake at dosages higher than the current recommended dietary allowance (RDA) for the prevention and/or reverse of immune system impairments.

***Zinc***

Zinc (Zn) acts as a powerful antioxidant and anti-inflammatory agent.^159^ It has a vital role in both innate and adaptive immune cells, because some cellular functions such as DNA replication, RNA transcription, cell division, and cell activation are Zn-dependent. Zn is also crucial for normal development and function of neutrophils and natural-killer cells.^160^ Zn deficiency leads to thymic atrophy, lymphopenia, impaired cellular and antibody-mediated immune responses and increases susceptibility to a variety of pathogens and infectious diseases.^161^

Some aspects of immunity can be suppressed by even marginal Zn deficiency, which is more common than severe Zn deficiency,^124^ particularly in the elderly due to their inadequate dietary intake.^162,163^ In addition, serum Zn levels decline with age.^164,165^ Several randomized controlled trials demonstrate that supplementation with low to moderate doses of Zn (ranging from 10 to 45 mg zinc/day) in healthy elderly individuals improves immune function, such as increased number of cytotoxic T lymphocytes and NK cells, reduced number of activated T helper cells, and lower incidence of infections.^166-168^ Velthuis et al^169^showed that increasing the intracellular Zn2+ concentration with zinc-ionophores like pyrithione (PT) can efficiently impair the replication of some RNA viruses, including influenza virus. In their study, it was demonstrated that the combination of Zn2+ and PT at low concentrations can inhibit the replication of SARS-CoV in cell culture.^169^ Another systematic review demonstrated that zinc supplementation was significantly associated with reducing rates of pneumonia and therefore, recommended supplementing zinc intake in deficient populations.^170^

Although several studies favor the vital role of Zn in immunity enhancement, on the other side, there are few preliminary studies,^171,172^ implying the possible adverse effects of Zn in cell cultures. For instance, Phillips et al reported that neurovirulent murine coronavirus JHM.SD uses cellular Zn metalloproteases for virus entry and cell-cell fusion.^171^ They found that inhibition of matrix metalloproteinase and metalloprotease (ADAM)-family Zn metalloproteases markedly decreased both entry and cell-cell fusion. They concluded that Zn metalloproteases must be considered potential contributors to coronavirus fusion. In this regard, it is worth-mentioning that ACE which COVID-19 as well as SARS-CoV were shown to use as its main entry receptor,^66^ belongs to the M2 Zn metalloproteinase family.^173^ Moreover, in the study of Tan et al, it was proposed that binding of the 50-untranslated region of coronavirus RNA to Zn finger CCHC-type and RNA-binding motif 1 enhances both viral replication and transcription.^172^ Therefore, in the light of current studies, it appears that adequate intake of Zn is essential for proper function of immune system; however, there might be some hazards in case of excessive Zn intake. Overall, precautions should be taken when it comes to supplementation of Zn for the prevention or treatment of infections.

***Iron***

Iron is an essential micronutrient for cellular energy metabolism, oxygen transport, and several enzymatic reactions.^174^ The iron-containing proteins are needed for crucial cellular and organismal actions including mitochondrial respiration, oxygen transport, cell signaling, nucleic acid replication and repair, and host defense.^175^ An adequate amount of iron is critical for multiple immune functions, including the differentiation and proliferation of T-cells and generation of reactive oxygen species (ROS) that kill infectious agents. However, iron is also required by most pathogens for replication and survival. During an acute inflammatory response, serum iron concentration depresses, while level of ferritin (the iron storage protein) increases, proposing that sequestration of free iron by host proteins prevents acquisition by pathogens^176^; this is called nutritional immunity, which is an important host response to infections.^177^

Iron ions serve as the catalytic portion of enzymes that mediate redox reactions in key cellular processes, like DNA replication and energy production.^178^ Fe (III) prevents replication of DNA and RNA viruses.^179^ During viral infection, ROS and superoxide hydrogen production may happen. Iron can be easily oxidized and reduced, making it necessary to be able to catalyze the exchange of hydrogen peroxides to free radicals.^180^Iron-deficient children and iron deficiency anemia has been reported as a risk factor for the development of recurrent acute respiratory tract infections.^181^ Furthermore, lactoferrin as the iron-binding glycoprotein shows inhibitory actions against a different range of viruses *in vitro*. Indeed, it was observed that lactoferrin consumption might protect the host from virus infections by preventing the virus attachment to the cells, the virus replication in the cells, and improvement of systemic immune function.^182^

Excessive iron can also be harmful, because of its ability to favor animal viral infections. The metal is required for host cell to synthesize virions which can weaken the cell function defense and raise oxidative stress. In humans and animal models, viral infections can lead to up-regulation of the iron-repressing defense system. Iron chelators are perfect candidates for use in co-infection and excess iron states because they have been effective in inhibition of HIV replication.^183^ Overall, it seems essential to restrict iron intake in times of either potential or existing infections to deprive the pathogens of iron, but to have an adequate intake based on RDA to maintain an optimum immune response and avoid the possibility of excess amounts of iron which may induce iron toxicity.^184^

***Selenium***

Selenium (Se) is an important trace element with anti-inflammatory and antioxidant effects.^185^ Low serum Se level has been correlated with poor immune function and increased risk of mortality. Adequate Se intake has antiviral effects.^186^ The patients diagnosed with influenza virus present a noticeable increase in protein, lipid, and DNA oxidation products in plasma and urine.^187-190^I n some types of RNA viruses, oxidative stress prompts fast mutation rates - frequently to virulence.^191^ Se deficiency impairs the innate and acquired immunity unfavorably influencing both cell-mediated immunity and humoral immunity (i.e., antibody production).^192,193^ Sequencing of virus genes isolated from Se-deficient and Se-adequate mice showed a strong effect of the Se status on virus mutation.^194^ Se deficiency also seems to enhance the virulence or progression of some viral infections.^195,196^ However, Se supplementation could ameliorate cell-mediated immunity in deficient individuals and enhance the immune response to viruses; on the other hand, Se supplementation may aggravate allergic asthma and weaken the immune response to parasites.^197,198^ Meanwhile, dramatic evidence demonstrates that Se plays a role in the regulation of cytokines and eicosanoids production that adjust the immune response.^199^ Overall, Se plays an important role in balancing the redox state, and helping to protect the host from oxidative stress induced by inflammatory reactions and anti-microbial effects of macrophages. Consequently, Se supplementation might be a helpful choice for the prevention or treatment of different types of viruses such as COVID-19 virus. However, Se status of the host is an important factor, when considering Se supplementation.^197^

### Water and electrolytes

It is usually recommended that higher fluid intake can prevent respiratory infections. Patient with COVID-19 symptoms or pneumonia can also benefit from higher intake of fluid unless contraindicated (such as edema or diarrhea). The recommended amount is usually 1 mL/kcal water or 2-3 L/d, being advised to drink between meals to prevent food reflux or aspirations.^27,28^

It was reported that patients with lower respiratory tract infections especially children are at risk of developing hyponatremia due to possible inappropriate antidiuretic hormone secretion (ADH) or excessive administration of free water or lower sodium intake.^200-202^ Moreover, as respiratory acidosis may occur following severe respiratory symptoms, serum electrolytes balance must be checked for hospitalized patients with COVID-19. The hypomagnesemia and hypophosphatemia should be avoided because magnesium and phosphorous both act as important intracellular buffers and contribute to the production of adenosine triphosphate (ATP). Their serum levels should be maintained at normal range, since high serum magnesium concentration (higher than 4-5 mmol/L) as well as severe hypophosphatemia (<1 mg/dL) can decrease respiratory function.^27,203-204^

### Phytochemicals

Plants provide us with a range of therapeutic metabolites that have the ability to prevent viral replication by controlling viral absorption, attaching to cell receptors, and inhibiting virus penetration into the host cell.^205^ Flavonoids are examples of metabolites which exhibit antiviral activity. Specially, luteolin, apigenin, quercetin, daidzein, amentoflavone, epigallocatechin, epigallocatechin gallate, gallocatechin gallate, puerarin, and kaempferol were found to hinder the SARS-CoV 3CL pro-proteolytic activity.^206-209^ Consequently, the antiviral effect is assumed to be specifically related to repress the SARS-CoV 3CLpro activity in some cases. Additionally, experimental study showed that isobavachalcone, quercetin 3-β-d-glucoside herbacetin, and helichrysetin were reported to block the MERS‐CoV 3CLpro enzymatic activity. Also, some flavonoid derivatives with carbohydrate or hydrophobic molecule in their core structures have been shown to yield a good inhibitory function.^210^

Resveratrol as a plant compound with antiviral activity was reported to stimulate ERK1/2 signaling pathway and activate cell proliferation and improve SIR1 signaling, which are associated with DNA repair and cellular survival, following DNA damage.^211-214^ Moreover, MERS-CoV infection can lead to the inflammatory cytokines production while, resveratrol might decrease the inflammation via preventing the NF-κB pathway.^215-216^ In a study by Lin et al, it was observed that after MERS-CoV infection, the cleaved caspase 3 levels were reduced by resveratrol.^215^They believed that the mentioned changes might be due to the caspase 3 cleavage direct inhibitions by reservation of the cell survival as well as the upstream event inhibition, necessary for caspase 3 cleavages or the decrease in virus-induced apoptosis by resveratrol.^217^

Overall, studies support the favorable effects of phytochemicals especially flavonoids and resveratrol in immunity enhancement following viral infections. Though further studies are warranted in case of antiviral impacts of phytochemicals, it appears that their adequate intake can contribute to boost the immune system, particularly in COVID-19.

### Probiotics 

Probiotics are “live strains of strictly selected microorganisms which, when administered in adequate amounts, confer a health benefit on the host”.^218^Common instances are the Lactobacilli and Bifidobacteria species. Ingested live probiotics can modulate immune functions through the interaction with several receptors on intestinal epithelial cells and other gut-associated immune cells, including M-cells and DCs.^219^ Probiotics have positive effects on gastrointestinal disorders and allergic diseases. Also, the effectiveness of probiotics for diseases treatment such as type 2 diabetes^220-221^ and obesity has been proven.^222^

Probiotics have effects on both the innate and acquired immune systems and have the potential to reduce the infections severity in the upper respiratory as well as gastrointestinal tracts.^223-225^ Studies have revealed that probiotics could prevent viral attachment through competitive inhibition, if they were able to bind to viral receptors at the cells surface.^226^In a study by Freitas et al, it was reported that a strain of *Bacteroides thetaiotaomicron* and the *Lactobacillus casei* strain DN114001 produce a compound that partly keep epithelial cells from the infection of rotavirus *in vitro* by modifying the cells apical glycosylation pattern.^227,228^ It is also probable that probiotics indirectly affect viruses through changing the cells state, and triggering innate and/or adaptive immunity.^223,229^ A study showed that *Enterococcus faecium* acts as beneficial antiviral agent by inhibiting transmissible gastroenteritis virus replication in swine testicle cells. The following overlapping mechanisms might lead to the detected fall in virus growth: inactivation or absorptive trapping of virus particles by the probiotic bacteria surface components, direct interfering with the attachment of virus and the stimulation of antiviral cytokines IL-6 and IL-8.^226^

Interestingly, the intestinal barrier can be reinforced by probiotics through the rise in mucins, the tight junction proteins and the Paneth and Goblet cells.^230^ The regeneration of mucosa is improved by mucin ability to inhibit the attachment of virus to epithelial cells and stop the virus replication. Probiotics also have the ability to modify the functions of epithelial cells, CD4+ CD8+ T lymphocytes DCs, NK cells, and induce secretory immunoglobulins synthesis, helping to deactivate a virus.^231,232^ Although the scientific evidence is too weak to favor the use of probiotics to reduce respiratory infections and improve vaccination response, especially in the elderly,^226-233^ probiotics are projected to be among the rational adjunctive choices for the treatment of several viral diseases.

## Conclusion

A summary of energy and macronutrients requirements for the management of COVID-19 is shown in Box 1. According to the studies reviewed, it is recommended to provide a relatively high-energy diet for patients afflicted with COVID-19 pneumonia, due to their increased metabolic demands. On the other hand, for un-afflicted persons, quarantined at home, we recommend to avoid sedentary lifestyle and excessive energy intake which can lead to the obesity and decreased immunity. Overall, avoiding undernutrition or over-nutrition by ensuring sufficient nutrients supply through healthy food preferences seems to be essential in case of COVID-19 prevention or treatment.

**Box 1.** Summary of energy and macronutrients requirement for the management of COVID-19

1. Notice regular nutrition assessment of patients considering anthropometry, laboratory, and clinical data to avoid malnutrition.
2. Design an individualized diet with energy intake of approximately 30 to 35 kcal/kg IBW and 1.2-1.5 g protein/kg IBW to meet hyper-metabolic requirements of patients with COVID-19 pneumonia.
3. Consider adequate amount of dietary fat (35%-40% of total calories) especially during ARDS and appropriate proportion of fatty acids (EFA, PUFA, and MUFA)
4. Prevent or correct dehydration using enough fluid intake (2–3 L/d) between meals, unless contraindicated.
5. Start PN when enteral nutrition has failed or during severe malabsorption.
6. Provide small, frequent feedings to reduce food reflux and aspirations.
7. Support lung function and prevent additional concomitant infections with higher intake of antioxidant and anti-microbial nutrients as discussed in detail through dietary sources or supplements, if needed (PN).
8. Plan a healthy diet during enteral feeding, considering adequate nutraceuticals intake including prebiotics, probiotics, and phytochemicals

Appropriate protein intake of 1.2 to 1.5 g/kg IBW (equal to 15%-20% of calories) with at least 50 percent from HBV proteins for adults is recommended to avoid more respiratory complications and support immunity during infection with COVID-19. However, diet should be individually planned, considering other comorbidities of patients. A balanced ratio of carbohydrates (40% to 50% of calories) mostly as the complex carbohydrates and adequate intake of functional fibers is critical in medical nutrition therapy of COVID-19 pneumonia.

Adequate intake of EFAs and appropriate PUFAs and MUFAs intake within the range of 30% to 45% of calories from dietary fat can be effective in the management of COVID-19 pneumonia with ARDS symptoms by decreasing CO2 production and ventilator dependency. Although omega 3 fatty acids are regarded as anti-inflammatory food agents, the evidence is not suggestive for their administration after viral infection. This is because they can modulate anti-viral CD8+ T cells and their downstream inflammatory cascades.

Patient with COVID-19 symptoms or pneumonia can also benefit from higher intake of fluid, unless contraindicated. The recommended amount is usually 1 mL/kcal water or 2-3 L/d. As respiratory acidosis may occur following severe respiratory symptoms, serum electrolytes balance, especially magnesium, phosphorous and sodium must also be checked.

A summary of micronutrients and nutraceuticals are presented in Table 1. It seems that VitD supplementation can be helpful both as a preventive strategy and an anti-ACE2 therapeutic agent in COVID-19 management to maintain its serum level at normal range, but avoid the vitamin toxicity. VitC can also help avoid and cure respiratory and systemic infections by enhancing the immunity. However, further studies are needed to confirm the potential effect of VitC on severe viral respiratory tract infections especially in case of the recent COVID-19 epidemic. As VitA has a wide range of immunological functions and contributes to the first-line defense of the body, it appears that VitA might be promising in the prevention and treatment of lung infections associated with COVID-19. However, due to potential adverse effects, VitA supplements should be administered for those with evidence of VAD.

The optimum intake of VitE required for better immunity function has not been reported, likely due to prior VitE status and the person’s comorbidities; though studies have reported effective immune dosage of 200–800 mg/d. Given the effects of VitB complex, especially B6, B9, and B12 on the immunity, further studies are warranted to evaluate the efficacy of VitBs intakes at higher dosages than recommended for the prevention and/or reverse of immune system impairments.

In terms of trace elements, it appears that adequate intake of Zn is essential for proper function of the immune system; however, there might be some health hazards in case of excessive Zn intake. Therefore, precautions should be taken when it comes to supplementation of zinc for the prevention or treatment of infections. It is also essential to restrict iron intake in times of either potential or existing infections, but to have an adequate daily intake, based on the RDA, to maintain an optimum immunity and prevention of viral infections. Moreover, evidence show that adequate selenium intake or its supplementation within safe dose might be promising in the prevention and treatment of respiratory infections including COVID-19.

Based on current limited studies, it seems that adequate intake of alpha linolenic acid, phytochemicals and probiotics may help boost the immune system and prevent or even treat viral infections.

Finally, the present review tried to help the health practitioners to effectively manage patients with COVID-19 by taking nutritional considerations more into account. However, further well-designed clinical trials are needed to confirm the efficacy of nutritional recommendations and determine their effective dose during the outbreak of respiratory viral infections.

## Acknowledgments

We wish to express our appreciation to the Research Vice-Chancellor of Tabriz and Maragheh Universities of Medical Sciences.

## Funding

The present comprehensive review was performed without any grant and just as the authors’ interest and responsibility.

## Competing interests

The authors declare no conflict of interest.

## Ethical approval

This review did not need any formal approval from ethical committee.

## Authors’ contributions

VEA, MSA and NLY contributed in designing the study, searching for resources and writing the manuscript. GP cooperated in writing the mechanism of action for nutrients. MSA, JRH, AO, and BB each contributed in the revision of the manuscript.

**Table 1:** Significant antiviral micronutrients or nutraceuticals

**Nutrient/Nutraceutical**	**RDAs for adults (≥19 years)**	**Major sources** ^b^
Vitamin D	19-70 years: 600 IU/day >71 years: 800 IU/day	Sunshine, salmon, fortified foods like milk
Vitamin A	Men: 900 µg/d Women: 700 µg/d	Liver, milk, egg, turkey meat, sweet potato, carrots, spinach
Vitamin E	15 mg/d	Vegetable oils especially sunflower and canola oil, nuts
Vitamin C	Men: 90 mg/d Women: 75 mg/d	Sweet pepper, citrus fruit, kiwifruit, strawberry
Vitamin B6	19-50 years: 1.3 mg/d ≥51 years: Men: 1.7 mg/d Women: 1.5 mg/d	Salmon, turkey, chicken, potato, banana
Vitamin B9	400 µg/d	Lentils, beans, spinach, asparagus, broccoli
Vitamin B12	2.4 µg/d	Liver, clams, beef, oyster, mackerel, skim milk
Selenium	55 µg/d	Brazil nuts, halibut, tuna, oysters, chicken, egg, rice
Zinc	Men: 11 mg/d Women: 8 mg/d	Oyster, beef, pork, chicken, dairy product, baked bean
Iron	Men: 8 mg/d Women: 19–50 years: 18 mg/d > 50 years: 8 mg/d	Clams, liver, beef, oyster, baked bean, fortified cereals
Omega 3 fatty acids^a^	ALA: Men: 1.6 g/d Women: 1.1 g/d DHA and EPA: ND	ALA: Flaxseed, canola oil, soybean oil, walnut, pumpkin seeds DHA & EPA: Cod liver oil, herring, salmon, sardines
Probiotics	ND	Fermented foods: yogurt, kefir, sauerkraut, pickles, tempeh
Prebiotics	ND	Oat, wheat, banana, onions, garlic, tomato, asparagus, chicory root
Phytochemicals (flavonoids, resveratrol, quercetin, etc.)	ND	Coffee, black tea, green tea, most of fresh fruits and vegetables like apple, citrus fruits, berries, grapes, onion and also some herbal plants

Abbreviations: RDA, recommended dietary allowance; ALA, ɑ-Linolenic acid; ^a^Based on Adequate Intakes (AIs) ^b^Source: Kathleen Mahan L, Raymond JL, eds. Krause's Food & the Nutrition Care Process. St. Louis, Missouri: Elsevier; 2016
